# Genetic variation in candidate obesity genes *ADRB2*, *ADRB3*, *GHRL*, *HSD11B1*, *IRS1*, *IRS2*, and *SHC1 *and risk for breast cancer in the Cancer Prevention Study II

**DOI:** 10.1186/bcr2114

**Published:** 2008-07-08

**Authors:** Heather Spencer Feigelson, Lauren R Teras, W Ryan Diver, Weining Tang, Alpa V Patel, Victoria L Stevens, Eugenia E Calle, Michael J Thun, Mark Bouzyk

**Affiliations:** 1 Department of Epidemiology and Surveillance Research, American Cancer Society, Williams Street, NW, Atlanta, Georgia 30303-1002, USA; 2 Center for Medical Genomics, Department of Human Genetics, Emory University School of Medicine, North Decatur Road, Atlanta, Georgia 30033 USA

## Abstract

**Introduction:**

Obesity has consistently been associated with postmenopausal breast cancer risk. Proteins that are secreted by adipose tissue or are involved in regulating body mass may play a role in breast tumor development.

**Methods:**

We conducted a nested case-control study among postmenopausal women from the American Cancer Society Cancer Prevention Study II Nutrition Cohort to determine whether genes associated with obesity increase risk for breast cancer. Tagging single nucleotide polymorphisms (SNPs) were selected to capture common variation across seven candidate genes that encode adipose-related proteins: *ADRB2*, *ADRB3*, *GHRL*, *HSD11B1*, *IRS1*, *IRS2*, and *SHC1*. Thirty-nine SNPs were genotyped in 648 cases and 659 controls. Logistic regression models were used to examine the association between each tagging SNP and risk for breast cancer while adjusting for matching factors and potential confounders. We also examined whether these SNPs were associated with measures of adult adiposity.

**Results:**

Two out of five tagging SNPs in *HSD11B1 *were associated with breast cancer (rs11807619, *P *= 0.006; rs932335, *P *= 0.0001). rs11807619 and rs932335 were highly correlated (*r*^2 ^= 0.74) and, when modeled as a haplotype, only haplotypes containing the rs932335 C allele were associated with breast cancer. The rs932335 C allele was associated with a nearly twofold increased risk for breast cancer (odds ratio = 1.83, 95% confidence interval = 1.01–3.33 for C/C versus G/G). Three of the 11 SNPs for *IRS2 *were associated with breast cancer (rs4773082, *P *= 0.007; rs2289046, *P *= 0.016; rs754204, *P *= 0.03). When these three SNPs were examined as a haplotype, only the haplotype that included the G allele of rs2289046 was associated with breast cancer (odds ratio = 0.76, 95% confidence interval = 0.63–0.92 for TGC versus CAT). *IRS2 *rs2289046, rs754204, and rs12584136 were also associated with adult weight gain but only among cases. None of the other SNPs in any gene investigated were associated with breast cancer or adiposity.

**Conclusion:**

Our findings suggest that these tagging SNPs in *HSD11B1 *and *IRS2 *mark regions of the genome that may harbor risk alleles for breast cancer, and these associations are probably independent of adiposity.

## Introduction

Obesity is a leading risk factor for postmenopausal breast cancer. High body mass index (BMI) and adult weight gain have consistently been associated with increased risk for breast cancer [1-3]. This increased risk is thought to result primarily from circulating estrogens derived from extraglandular aromatization of plasma androstenedione to estrone in adipose tissue [4,5]. However, adipose tissue is also an active endocrine and metabolic organ that can have far-reaching effects on the physiology of other tissues [2]. Therefore, proteins that are secreted by adipose tissue or are involved in regulating body mass may play a role in breast tumor development through mechanisms independent from estrogen production.

To examine whether genes that encode adiposity-related proteins influence the risk for breast cancer, we conducted a nested case control study among predominantly white, postmenopausal women from the American Cancer Society Cancer Prevention Study II Nutrition Cohort. Seven candidate genes were chosen for inclusion in this study: *ADRB2*, *ADRB3*, *GHRL*, *HSD11B1*, *IRS1*, *IRS2*, and *SHC1*. Although many more genes are related to obesity and energy balance, we chose candidate genes that had previously been associated with at least one measure of obesity and with risk for breast cancer [6-10] or other cancer [11,12], or had demonstrated activity in breast cancer cell lines [13-15].

## Materials and methods

### Study population

Cases and controls included in the present study were drawn from the Cancer Prevention Study II Nutrition Cohort, a prospective study of cancer incidence that began in 1992 and includes approximately 184,000 US adults. The characteristics of this cohort are described in detail elsewhere [16]. Blood samples were collected from a subgroup of 39,376 cohort members (*n *= 21,965 women) between June 1998 and June 2001. After obtaining written informed consent, a maximum of 43 ml nonfasting whole blood was collected from each participant and samples were separated into aliquots and frozen in liquid nitrogen vapor phase at approximately -130°C for long-term storage. The women who provided blood specimens were similar to the overall population in the distribution of most demographic and lifestyle characteristics. This study has been approved by the Institutional Review Board of Emory University and is in compliance with the Helsinki Declaration.

Breast cancer patients (cases) were initially identified based on responses to cancer history questions on surveys sent to cohort participants in 1997, 1999, 2001, and 2003. These cancers were subsequently verified through medical records, linkage with state cancer registries, or death certificates. The cases were matched to cancer-free (except nonmelanoma skin cancer), female Nutrition Cohort control individuals (controls) for single year of age (± 6 months), race/ethnicity (white, African American, Hispanic, Asian, or other/unknown), and date of blood collection (within 6 months).

### Single nucleotide polymorphism selection and genotyping

The selection of specific single nucleotide polymorphisms (SNPs) for this study involved several steps. The list of candidate SNPs was created based on the following criteria: inclusion in the International HapMap Project database [17]; location within 10 kilobases (kb) of one of the candidate genes (to capture potential regulatory regions) as well as all of the exons and introns; and minor allele frequency of at least 5% in a Caucasian population (to ensure sufficient power). Also included were three nonsynonymous SNPs listed on dbSNP [18] that were not in HapMap but had been validated and had a minor allele frequency of above 5% in a Caucasian population (*ADRB3 *rs4994, *IRS1 *rs1801276, and *IRS2 *1805097).

These criteria yielded a total of 72 SNPs among the seven genes in this analysis. From this list, tagging were selected using the Tagger program in Haploview (v.3.32) [19,20]. Tagger is a computer program that is used to select and evaluate tagging SNPs based on the empirical patterns of linkage disequilibrium (LD) called 'bins'. This allows common variation across the region of interest to be captured with fewer SNPs. For this analysis, we used pair-wise tagging to choose SNPs that were correlated at *r*^2 ^equal to 0.80 or greater with all other SNPs in a LD bin. Furthermore, we required that SNPs previously shown to be associated with cancer and nonsynonymous SNPs be 'forced in' as tagging SNPs. To further reduce the number of SNPs, 27 singleton SNPs (SNPs that were not in LD with any other SNPs) located more than 1 kb from an exon were excluded (singleton SNPs from bins in or near to exons were not excluded). This resulted in a total of 45 SNPs (including the three nonsynonymous SNPs described above) selected. Thirty-nine SNPs were successfully genotyped in the first round. The remaining six failed after two independent attempts, possibly because of high GC content and/or low-complexity in the SNP region.

Genotyping was performed by the Center for Medical Genomics at Emory University using the Beckman SNPstream genotyping system. SNPstream is designed to conduct high-throughput, multiplex genotyping using single-base primer extension technology in a tagged fluorescent assay. We used a SNPstream companion primer design website [21] to design three primers (two for PCR and one for single-base extension) per SNP and multiplex them into 48-plex primer panels. The PCR primers were used to amplify an approximately 100 base pair region flanking each SNP in a 384-well PCR plate. Image processing and genotype calling were carried out using the GenomeLab SNPstream Genotyping System Software Suite v2.3. Details of the primer sequences and experimental protocol are available upon request.

Laboratory personnel were blinded to case-control status. Positive and negative DNA controls were included on each plate and 10% blind duplicates were randomly interspersed among the case-control samples to validate genotyping procedures. Concordance among duplicates samples was above 99%. The overall call rate for each genotyping assay ranged from 87.4% to 99.6%. The two lowest call rates were for *IRS2 *rs2289046 (87.4%) and *IRS2 *rs4773092 (92.5%). We carefully evaluated these assays, checked the concordance of duplicate samples, and tested for deviations from Hardy-Weinberg equilibrium. We found no reason to exclude them from the analysis. One SNP (*ADRB2 *rs1042714) was dropped from the analysis because the allele distribution among controls deviated from Hardy-Weinberg equilibrium (*P *= 0.004). No other deviations from Hardy-Weinberg equilibrium were observed (at the *P *< 0.01 level).

### Statistical analysis

Unconditional logistic regression models were used to examine the association between each SNP and breast cancer while controlling for matching factors. For each SNP, homozygotes for the more common allele were used as the reference group. Using questionnaire data collected before diagnosis, we evaluated BMI (weight [kg]/height [m]^2^), adult weight change (from age 18 years), postmenopausal hormone (PMH) use (never, estrogen replacement therapy, combination hormone therapy, or missing), family history of breast cancer (mother or sister [s] with breast cancer), and history of breast cysts as possible confounders. BMI and weight gain were modeled as continuous variables. We also tested for effect modification between the genotypes and BMI, postmenopausal hormone use, family history of breast cancer, and history of breast cysts using likelihood ratio tests. Models with the main effect of genotype and the covariate of interest were compared with models with the main effects of genotype and the covariate of interest plus a multiplicative interaction term of the two variables.

Among SNPs that were statistically significant and in strong LD, we conducted haplotype analysis using the program TAGSNPS [22] to estimate haplotype frequencies. The TAGSNPS program uses an expectation maximization approach to assign haplotypes based on unphased genotype data. We used logistic regression models with the most common haplotype as the reference to estimate haplotype-specific odds ratios (ORs) while controlling for matching factors and other covariates.

Finally, we used age-adjusted linear regression models to examine whether any of these SNPs were associated with self-reported BMI at age 18 years, BMI in 1992, or adult weight change. Age-adjusted means for each metric were computed by genotype.

## Results

A total of 648 cases and 659 controls were included in the analysis. Characteristics of the study population are shown in Table 1. The study population was 99% white, and the mean age at breast cancer diagnosis was 68.9 years. Cases were slightly more likely than controls to have a BMI of 30.0 kg/m^2 ^or greater (16.4% versus 15.6%), but this difference was not statistically significant (*P *= 0.16). Cases and controls reported similar weight change since age 18 years (27.9 lb [12.7 kg] versus 27.1 lb [12.3 kg]; *P *= 0.58). It is well established (in this cohort [23] and others [1,24]) that an interaction exists between obesity and PMH use, in which the deleterious effect of obesity is observed only among women not using PMH. The apparent lack of association in this analysis can be accounted for by this relationship. Sixty per cent of the study population use PMH; among those women not using PMH, obesity is associated with increased risk for breast cancer.

**Table 1:** Characteristics of the breast cancer cases and controls

Variable	Cases (*n *= 648)	Controls (*n* = 659)	*P *diff^a^
Age at blood draw (years; mean ± SD)	69.2 (5.8)	69.3 (5.76)	*P *= 0.93
Age at diagnosis (years; mean ± SD)	68.9 (6.25)	-	-
BMI (kg/m^2^; mean ± SD)	25.2 (4.54)	25.5 (4.56)	*P *= 0.22
Adult weight change (lb; mean ± SD)	27.9 (25.65)	27.1 (25.91)	*P *= 0.58
Race (*n *[%])			
White	642 (99.1)	653 (99.1)	*P *= 0.98
Other	6 (0.9)	6 (0.9)	
BMI (kg/m^2^; *n *[%])			
<25	364 (56.2)	338 (51.3)	*P *= 0.16
25–30	170 (26.2)	210 (31.9)	
30+	106 (16.4)	103 (15.6)	
Missing	8 (1.2)	8 (1.2)	
History of breast cysts (*n *[%])			
No	402 (62)	460 (69.8)	*P *= 0.003
Yes	246 (38)	199 (30.2)	
Postmenopausal hormone use (*n *[%])			
Never	213 (32.9)	255 (38.7)	*P *< 0.0001
ERT	204 (31.5)	260 (39.5)	
CHRT	203 (31.3)	120 (18.2)	
Unknown	28 (4.3)	24 (3.6)	
Family history of breast cancer (*n *[%])			
No	513 (79.2)	564 (85.6)	*P *= 0.002
Yes	135 (20.8)	95 (14.4)	
Education (*n *[%])			
<High school	7 (1.1)	25 (3.8)	*P *= 0.0008
High school	166 (25.6)	196 (29.7)	
Some college	181 (27.9)	190 (28.8)	
College +	294 (45.4)	248 (37.6)	
Age at menopause (years; *n *[%])			
<40	55 (8.5)	72 (10.9)	*P *= 0.002
40 to 44	65 (10)	85 (12.9)	
45 to 49	139 (21.5)	179 (27.2)	
50 to 54	301 (46.5)	258 (39.2)	
55+	88 (13.6)	65 (9.9)	

^a^*P *value is a *t*-test for the means, and a Pearson χ^2 ^for the frequencies. BMI, body mass index; CHRT, combined hormone replacement therapy; ERT, estrogen replacement therapy; SD, standard deviation.

**Figure 1:**
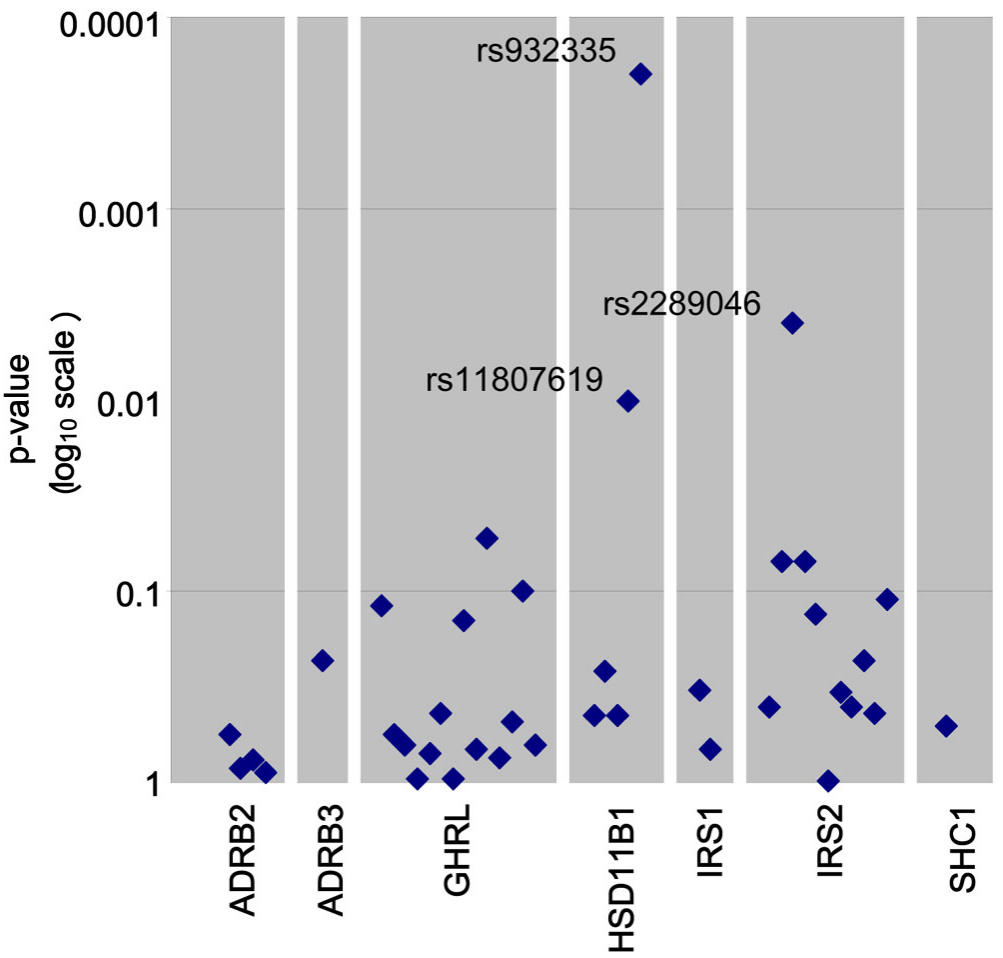
Log-additive model *P *values for the association between breast cancer and 38 SNPs in seven genes. SNP, single nucleotide polymorphism.

**Figure 2:**
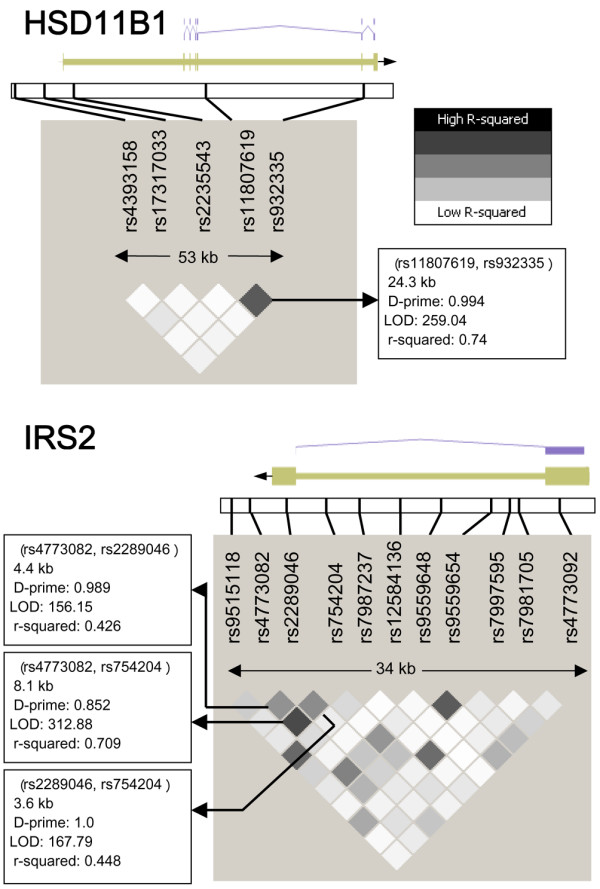
Pattern of linkage disequilibrium for tagging SNPs genotyped in *HSD11B1 *and *IRS2*. The gray shaded boxes correspond to the paired *r*^2 ^between the single nucleotide polymorphisms (SNPs). The associated protein products are also shown.

Figure 1 shows the log-additive *P *values for the association between breast cancer and all 38 SNPs analyzed. Table 2 shows the association between breast cancer and tagging SNPs in *HSD11B1*. In multivariate adjusted models, SNPs rs11807619 and rs932335 were both associated with breast cancer. For rs11807619, the T allele was associated with a 40% increased risk for breast cancer (OR = 1.41, 95% CI = 1.10 to 1.80 among women with any T allele versus G/G women; *P *= 0.006). For rs932335, the adjusted ORs were 1.56 (95% CI = 1.22 to 2.00) for G/C and 1.83 (95% CI = 1.01 to 3.33) for C/C compared with G/G (*P *for trend = 0.0002). Further examination of these SNPs using Haploview indicated that they were highly correlated (D' = 0.99 and *r*^2 ^= 0.74; Figure 2). Haplotype analysis of these two SNPs showed that rs11807619 was no longer an independent predictor of risk. The two haplotypes that carry the C allele of rs932335 were both associated with breast cancer.

**Table 2:** *HSD11B1 *SNP and haplotype associations with breast cancer

				Matching adjusted^a^			Multivariate adjusted^b^		
SNP	Alleles	Cases	Controls	OR	(95% CI)	*P *value^c^	OR	(95% CI)	*P *value^c^
*HSD11B1*	rs4393158								
	G/G	563	578	1.00	(-)		1.00	(-)	
	G/A	65	61	1.10	(0.76–1.59)		1.09	(0.75–1.59)	
	A/A	2	0	-	(-)	0.39	-	(-)	0.44
	G/A or A/A	67	61	1.14	(0.79–1.64)	0.5	1.13	(0.77–1.64)	0.55
	rs17317033								
	A/A	502	493	1.00	(-)		1.00	(-)	
	A/C	100	126	0.78	(0.58–1.04)		0.77	(0.57–1.04)	
	C/C	9	4	2.16	(0.66–7.09)	0.37	1.75	(0.52–5.83)	0.26
	A/C or C/C	109	130	0.82	(0.62–1.09)	0.18	0.80	(0.60–1.07)	0.14
	rs2235543								
	C/C	489	510	1.00	(-)		1.00	(-)	
	C/T	143	128	1.17	(0.89–1.53)		1.17	(0.89–1.54)	
	T/T	11	13	0.88	(0.39–1.99)	0.44	0.90	(0.39–2.05)	0.44
	C/T or T/T	154	141	1.14	(0.88–1.48)	0.32	1.14	(0.88–1.49)	0.33
	rs11807619								
	G/G	419	471	1.00	(-)		1.00	(-)	
	G/T	197	159	1.39	(1.08–1.78)		1.41	(1.10–1.82)	
	T/T	22	18	1.34	(0.71–2.55)	0.012	1.37	(0.71–2.63)	0.01
	G/T or T/T	219	177	1.38	(1.09–1.76)	0.008	1.41	(1.10–1.80)	0.006
	rs932335								
	G/G	349	421	1.00	(-)		1.00	(-)	
	G/C	234	185	1.52	(1.20–1.94)		1.56	(1.22–2.00)	
	C/C	30	20	1.75	(0.98–3.15)	0.0003	1.83	(1.01–3.33)	0.0002
	G/C or C/C	264	205	1.55	(1.23–1.95)	0.0002	1.59	(1.25–2.02)	0.0001

Haplotypes of rs11807619 and rs932235	GG	488.7	532.9	1.00	(-)		1.00	(-)	
	TC	120.3	97.3	1.35	(1.09–1.66)	0.0055	1.37	(1.11–1.70)	0.0040
	GC	33.4	24.2	1.54	(1.04–2.28)	0.030	1.62	(1.08–2.41)	0.019
	TG	0.6	0.6	0.89	(0.06–14.39)	0.93	0.96	(0.06–15.81)	0.98
				Global *P *value = 0.01			Global *P *value = 0.006		

^a^Adjusted for race, birth date, and blood draw date. ^b^Adjusted for race, birth date, blood draw date, adult weight change, breast cysts, family history of breast cancer, and hormone replacement therapy use. ^c^For *HSD11B1 P *values are provided for the log-additive model, and the dominant model; for Haplotypes of rs11807619 and rs932235, haplotype *P *values are compared with the most common haplotype. CI, confidence interval; *HSD11B1*, 11β hydroxysteroid dehydrogenase type 1 gene; OR, odds ratio.

Results for *IRS2 *SNPs are shown in Table 3. Three of the 11 tagging SNPs for *IRS2 *were associated with breast cancer (rs4773082, *P *= 0.007; rs2289046, *P *= 0.016; and rs754204, *P *= 0.03). The C allele of rs4773082 was associated with increased risk for breast cancer (OR = 1.41, 95% CI = 1.10 to 1.81 for T/C and C/C versus T/T). For rs2289046 the G allele was associated with a reduced risk for breast cancer; the adjusted ORs were 0.79 (95% CI = 0.61 to 1.01) for A/G and 0.52 (95% CI = 0.32 to 0.84) for G/G compared with A/A. The T allele of rs754204 was associated with increased risk for breast cancer (OR = 1.32, 95% CI = 1.03 to 1.70 for T/C and T/T versus C/C). These SNPs span a region of 8.1 kb, with D' ranging from 1.0 to 0.85 and *r*^2 ^ranging from 0.71 to 0.42 (Figure 2). Thus, we created a haplotype of these three SNPs. The TGC haplotype is associated with decreased risk (*P *= 0.0018), and the magnitude of the effect is similar to that of single SNP results for rs2289046.

**Table 3:** *IRS2 *SNP and haplotype associations with breast cancer

				Matching adjusted^a^			Multivariate adjusted^b^		
SNP	Alleles	Cases	Controls	OR	(95% CI)	*P *value^c^	OR	(95% CI)	*P *value^c^
*IRS2*	rs9515118								
	G/G	386	404	1.00	(-)		1.00	(-)	
	G/C	201	201	1.06	(0.83–1.34)		1.07	(0.83–1.37)	
	C/C	28	23	1.28	(0.72–2.29)	0.43	1.28	(0.70–2.31)	0.40
	G/C or C/C	229	224	1.08	(0.85–1.36)	0.54	1.09	(0.86–1.38)	0.49
	rs4773082								
	T/T	157	205	1.00	(-)		1.00	(-)	
	T/C	327	299	1.43	(1.10–1.86)		1.46	(1.12–1.91)	
	C/C	156	146	1.40	(1.03–1.91)	0.024	1.31	(0.96–1.80)	0.07
	T/C or C/C	483	445	1.42	(1.11–1.82)	0.005	1.41	(1.10–1.81)	0.007
	rs2289046								
	A/A	275	248	1.00	(-)		1.00	(-)	
	A/G	245	284	0.78	(0.61–0.99)		0.79	(0.61–1.01)	
	G/G	32	57	0.51	(0.32–0.81)	0.0018	0.52	(0.32–0.84)	0.004
	A/G or G/G	277	341	0.73	(0.58–0.92)	0.009	0.74	(0.58–0.94)	0.016
	rs754204								
	C/C	155	198	1.00	(-)		1.00	(-)	
	C/T	328	315	1.33	(1.02–1.73)		1.32	(1.01–1.73)	
	T/T	159	142	1.43	(1.05–1.94)	0.022	1.32	(0.96–1.82)	0.07
	C/T or T/T	487	457	1.36	(1.06–1.74)	0.015	1.32	(1.03–1.70)	0.03
	rs7987237								
	C/C	470	491	1.00	(-)		1.00	(-)	
	C/T	156	149	1.10	(0.85–1.43)		1.17	(0.90–1.53)	
	T/T	15	11	1.50	(0.68–3.31)	0.27	1.56	(0.70–3.50)	0.13
	C/T or T/T	171	160	1.13	(0.88–1.45)	0.35	1.20	(0.93–1.55)	0.17
	rs12584136								
	C/C	579	588	1.00	(-)		1.00	(-)	
	C/A	54	56	0.98	(0.66–1.44)		0.96	(0.64–1.43)	
	A/A	4	3	1.31	(0.29–5.88)	0.96	1.44	(0.31–6.71)	0.98
	C/A or A/A	58	59	0.99	(0.68–1.45)	0.97	0.98	(0.67–1.45)	0.93
	rs9559648								
	C/C	269	306	1.00	(-)		1.00	(-)	
	C/T	304	268	1.29	(1.02–1.62)		1.30	(1.03–1.65)	
	T/T	66	73	1.02	(0.71–1.49)	0.27	0.97	(0.66–1.42)	0.34
	C/T or T/T	370	341	1.23	(0.99–1.53)	0.07	1.23	(0.98–1.54)	0.07
	rs9559654								
	G/G	301	317	1.00	(-)		1.00	(-)	
	G/A	248	250	1.05	(0.83–1.33)		1.05	(0.82–1.34)	
	A/A	67	59	1.19	(0.81–1.75)	0.39	1.19	(0.80–1.76)	0.40
	G/A or A/A	315	309	1.07	(0.86–1.34)	0.52	1.08	(0.86–1.35)	0.52
	rs7997595								
	C/C	434	453	1.00	(-)		1.00	(-)	
	C/G	161	170	0.99	(0.77–1.28)		1.02	(0.79–1.32)	
	G/G	19	9	2.24	(1.00–5.01)	0.34	2.31	(1.02–5.24)	0.23
	C/G or G/G	180	179	1.05	(0.82–1.35)	0.68	1.09	(0.84–1.40)	0.50
	rs7981705								
	C/C	435	470	1.00	(-)		1.00	(-)	
	C/T	190	164	1.25	(0.98–1.60)		1.17	(0.91–1.51)	
	T/T	17	19	0.99	(0.50–1.93)	0.16	0.90	(0.45–1.79)	0.43
	C/T or T/T	207	183	1.23	(0.97–1.56)	0.09	1.14	(0.89–1.46)	0.29
	rs4773092								
	G/G	152	170	1.00	(-)		1.00	(-)	
	G/A	333	303	1.24	(0.95–1.62)		1.21	(0.92–1.60)	
	A/A	132	118	1.28	(0.92–1.78)	0.12	1.30	(0.93–1.82)	0.11
	G/A or A/A	465	421	1.25	(0.97–1.62)	0.08	1.24	(0.95–1.61)	0.11

Haplotypes of rs4773082, rs2289046, and rs754204	CAT	296.8	273.2	1.00	-		1.00	-	
	TGC	188.8	227.4	0.74	(0.62–0.90)	0.0018	0.76	(0.63–0.92)	0.0050
	TAC	107.3	104.6	0.99	(0.78–1.24)	0.89	1.04	(0.82–1.32)	0.75
	Other groups	52.2	49.9	0.98	(0.72–1.33)	0.88	0.96	(0.70–1.32)	0.80
				Global *P *value = 0.015			Global *P *value = 0.030		

^a^Adjusted for race, birth date, and blood draw date. ^b^Adjusted for race, birth date, blood draw date, adult weight change, breast cysts, family history of breast cancer, and hormone replacement therapy use. ^c^For *IRS2 P *values are provided for the log-additive model, and the dominant model; for Haplotypes of rs4773082, rs2289046, and rs754204, haplotype *P *values are compared with the most common haplotype. CI, confidence interval; *IRS2*, insulin receptor substrate 2 gene; OR, odds ratio.

No other SNPs in any of the other five genes were associated with breast cancer (Figure 1 and Additional file 1), and neither was there any evidence of effect modification by BMI, PMH use, family history of breast cancer, or history of breast cysts for any gene or SNP in this analysis (data not shown). None of our results for associations with breast cancer change if BMI is used in the model in place of weight change; adding BMI at age 18 years also does not change any associations with breast cancer. It should be noted that only one of the tested SNPs (rs932335 in *HSD11B1*) would be statistically significant after adjusting for multiple testing using the Bonferroni method.

Table 4 shows mean BMI and adult weight change by genotype for the *IRS2 *SNPs separately for cases and controls. We did not find any associations among our control women. Among the cases, the G allele for *IRS2 *rs2289046, which was protective for breast cancer, also was associated with less weight gain in adulthood compared to the A allele (*P *= 0.016). The T allele of rs754204, which was associated with increased risk for breast cancer, was associated with greater adult weight gain compared with the C allele (*P *= 0.012). The A allele of rs12584136 was associated with greater weight gain (*P *= 0.026), but was not associated with breast cancer. None of these SNPs would be statistically significant after adjusting for multiple testing using the Bonferroni method. None of the SNPs were associated with BMI. None of the SNPs in any of the other genes examined, including *HSD11B1*, were associated with adult weight change or BMI (data not shown), and neither were any of the SNPs associated with self-reported BMI at age 18 years (data not shown).

**Table 4:** Mean body mass index and adult weight change by *IRS2 *SNPs

**IRS2 SNP**		Cases				Controls			
		
		BMI		Adult weight change^a^		BMI		Adult weight change^a^	
		Mean	(SE)	Mean	(SE)	Mean	(SE)	Mean	(SE)
rs9515118	G/G	24.98	(0.23)	26.92	(1.33)	25.48	(0.22)	26.48	(1.29)
	G/C	25.66	(0.32)	30.94	(1.85)	25.57	(0.32)	27.6	(1.83)
	C/C	25.23	(0.85)	25.36	(4.90)	25.57	(0.98)	33.07	(5.64)
	*P *trend	0.16		0.29		0.82		0.31	
rs4773082	T/T	25.41	(0.37)	26.56	(2.11)	25.12	(0.32)	25.87	(1.81)
	T/C	25.2	(0.25)	27.81	(1.44)	25.64	(0.27)	27.43	(1.51)
	C/C	25.17	(0.37)	29.77	(2.07)	25.8	(0.38)	28.07	(2.16)
	*P *trend	0.64		0.28		0.15		0.42	
rs2289046	A/A	25.49	(0.28)	30.71	(1.55)	25.54	(0.29)	27.5	(1.65)
	A/G	24.97	(0.29)	26.09	(1.66)	25.52	(0.27)	27.06	(1.52)
	G/G	25.18	(0.82)	22.46	(4.60)	24.98	(0.60)	22.68	(3.39)
	*P *trend	0.28		0.016		0.53		0.31	
rs754204	C/C	24.99	(0.37)	24.42	(2.10)	25.13	(0.32)	25.45	(1.84)
	C/T	25.2	(0.25)	27.65	(1.43)	25.57	(0.26)	27.39	(1.46)
	T/T	25.55	(0.36)	31.77	(2.04)	25.9	(0.38)	28.38	(2.18)
	*P *trend	0.28		0.012		0.12		0.29	
rs7987237	C/C	25.17	(0.21)	28.3	(1.20)	25.58	(0.21)	27.24	(1.17)
	C/T	25.44	(0.37)	26.95	(2.09)	25.24	(0.38)	25.89	(2.13)
	T/T	25.1	(1.18)	25.73	(6.66)	25.51	(1.44)	34.45	(8.16)
	*P *trend	0.62		0.51		0.48		0.94	
rs12584136	C/C	25.16	(0.19)	27.36	(1.08)	25.54	(0.19)	27.02	(1.07)
	C/A	26.36	(0.63)	35.86	(3.56)	24.97	(0.61)	25.58	(3.49)
	A/A	25.52	(2.28)	34.05	(12.85)	30.06	(2.60)	57.32	(14.83)
	*P *trend	0.1		0.026		0.95		0.61	
rs9559648	C/C	25.34	(0.28)	27.65	(1.59)	25.38	(0.26)	26.05	(1.48)
	C/T	25.26	(0.26)	28.86	(1.49)	25.61	(0.28)	27.84	(1.59)
	T/T	24.61	(0.57)	25.55	(3.20)	25.64	(0.53)	27.16	(3.02)
	*P *trend	0.34		0.87		0.54		0.53	
rs9559654	G/G	25.05	(0.26)	26.14	(1.49)	25.68	(0.26)	27.39	(1.46)
	G/A	25.2	(0.29)	28.47	(1.64)	25.19	(0.29)	26.63	(1.64)
	A/A	25.74	(0.56)	31.38	(3.15)	25.64	(0.59)	25.8	(3.36)
	*P *trend	0.3		0.1		0.47		0.62	
rs7997595	C/C	25.38	(0.22)	29.46	(1.25)	25.39	(0.21)	26.26	(1.21)
	C/G	24.83	(0.36)	25.5	(2.05)	25.83	(0.35)	28.77	(2.00)
	G/G	25.58	(1.04)	25.76	(5.94)	25.91	(1.58)	33.47	(9.08)
	*P *trend	0.38		0.11		0.27		0.2	
rs7981705	C/C	25.2	(0.22)	27.1	(1.24)	25.3	(0.21)	26.3	(1.19)
	C/T	25.2	(0.33)	29.07	(1.88)	25.84	(0.35)	28.28	(2.03)
	T/T	26.24	(1.10)	34.9	(6.22)	27.62	(1.04)	32.52	(5.90)
	*P *trend	0.63		0.18		0.026		0.22	
rs4773092	G/G	25.25	(0.38)	8.65	(2.14)	25.66	(0.35)	26.36	(2.01)
	G/A	25.22	(0.25)	28.19	(1.42)	25.3	(0.26)	26.78	(1.52)
	A/A	25.28	(0.40)	27.03	(2.26)	25.85	(0.42)	28.5	(2.41)
	*P *trend	0.96		0.61		0.86		0.52	

All results are age-adjusted. ^a^Adult weight change is measured in pounds (lbs) from the weight at age 18 years to the weight in 1992. BMI, body mass index; *IRS2*, insulin receptor substrate 2 gene; SE, standard error.

## Discussion

Our results suggest that common variation in *HSD11B1 *and *IRS2 *may be associated with breast cancer among postmenopausal women. These associations appear to be independent of adiposity. The *HSD11B1 *rs932335 minor allele (allele frequency = 18% among controls) was associated with a nearly twofold increased risk for breast cancer (*P *= 0.0002). This SNP is highly correlated with rs11807619, and in haplotype analysis only the two common haplotypes that carried the rs932335 C allele were associated with risk for breast cancer. Three SNPs in *IRS2 *were associated with breast cancer and were also found to be in LD (rs4773082, rs2289046, and rs754204). When these SNPs were modeled as a haplotype, the haplotype that included the G allele of rs2289046 (allele frequency = 34% among controls) was the only common haplotype associated with breast cancer (OR = 0.76, 95% CI = 0.63 to 0.92 for one copy of TGC versus CAT; *P *= 0.0050). Thus, these tagging SNPs may mark regions of the genome that harbor risk alleles for breast cancer.

*HSD11B1 *is located at 1q32-41 and encodes the enzyme 11β-hydroxysteroid dehydrogenase type 1, which converts the inactive steroid cortisone into the active glucocorticoid cortisol, principally in the liver and adipose tissue [25]. To our knowledge, *HSD11B1 *has not been examined previously in any candidate gene study. However, 11 SNPs in *HSD11B1 *were included in a recent genome-wide scan of breast cancer [26,27] and none were associated with breast cancer, including SNPs that were highly correlated with ours. Two SNPs, rs4393158 and rs2235543, included in the scan, were also genotyped in our study with similar (negative) results.

Polymorphisms in *HSD11B1 *have been associated with type 2 diabetes and hypertension [25]. Recently, *HSD11B1 *was identified from a genome-wide linkage analysis as a possible candidate gene for weight change [28]. However, others [29] have reported no association with *HSD11B1 *and body composition. *HSD11B1 *variation has also been associated with polycystic ovarian syndrome [25], and ovarian steroids have been shown to regulate 11β-hydroxysteroid dehydrogenase expression in experimental models [13].

IRS2, a member of the insulin receptor substrate family, is expressed in nearly all cells and tissues. IRS2 regulates body weight control and glucose homeostasis [11]. Although the primary role played by IRS2 is in insulin signaling, IRS2 can also act as a substrate for insulin-like growth factor receptor and thus it acts in insulin-like growth factor signal transduction. Evidence also suggests that IRS proteins may play a role in estrogen signaling [8]. Previous studies of *IRS2 *variation and measures of adiposity have been inconsistent. Lautier and coworkers [30] suggested that *IRS2 *haplotypes were associated with severe obesity (BMI ≥40 kg/m^2^) among French individuals, but a later report by Sweeney and colleagues [31] did not confirm this association among US white and Hispanic women. Genome-wide linkage studies of obesity and BMI have not identified *IRS2 *as an important locus; however, the 13q region near *IRS2 *showed some evidence of linkage to BMI in a recent meta-analysis [32]. We observed modest associations between three *IRS2 *SNPs and adult weight change among our cases. Because these associations are not seen among the controls, we suspect that these results are due to chance. Furthermore, we do not think that the relationship between *IRS2 *and weight change explains the association between *IRS2 *SNPs and breast cancer. As shown in Table 3, the associations between these SNPs and breast cancer change very little, if at all, when adult weight change is included in the multivariate models. This suggests that the associations we observed with breast cancer are not mediated through increasing adiposity.

At least two previous studies have examined whether *IRS2 *SNPs are associated with breast cancer, but neither found an association [8,10]. The first study [10] sequenced coding regions to identify variants of interest, and then examined two *IRS2 *SNPs (rs4773092 and rs1805097) in a familial breast cancer study. Slattery and coworkers [8] used a large case-control study, including both white and Hispanic women, to examine the association between breast cancer and rs1805097 (G1057D), a SNP that had previously been associated with both colon cancer [11] and obesity [30]. We did not find an association between rs4773092 and postmenopausal breast cancer, and were unable to genotype rs1805097 because of assay failure. Data available from the Cancer Genetic Markers of Susceptibility initiative [27] indicates that one SNP in *IRS2 *is marginally associated with breast cancer (rs12584136, *P *= 0.051). This SNP was not associated with risk in our data. None of the other *IRS2 *SNPs genotyped in Cancer Genetic Markers of Susceptibility were associated with breast cancer, including those that were highly correlated with SNPs in our study.

It should be noted that we might have missed an association with breast cancer and the 27 singleton SNPs that we excluded from this study. Seven of these were in *HSD11B1*, six each in *ADRB2 *and *IRS2*, and four each in *GHRL *and *IRS1*. Singleton SNPs, by definition, are not correlated with any other SNPs that have been genotyped in the region. We chose to exclude singleton SNPs that were not near an exon, because we reasoned that they were likely to be less informative than SNPs that were correlated with other nearby SNPs, or SNPs that were located in or near an exon.

The strengths of this study include the population-based design and the use of tagging SNPs to conduct an effective survey of the variation in these genes. The primary limitation of this study is its relatively small sample size. Although these data suggest that *HSD11B1 *and *IRS2 *may be associated with risk for breast cancer, it is also possible that these findings are due to chance. If these findings are confirmed in subsequent studies, then additional genotyping in these regions is warranted.

## Conclusion

We conducted a nested case-control study among 648 cases and 659 controls to determine whether germline variation in seven genes that encode adipose-related proteins was associated with breast cancer. Our results suggest that common variation in *HSD11B1 *and *IRS2 *may be associated with breast cancer among postmenopausal white women. The *HSD11B1 *rs932335 minor allele (allele frequency = 18% among controls) was associated with a nearly twofold increased risk for breast cancer (*P *= 0.0002). Three SNPs in *IRS2 *were also associated with breast cancer (rs4773082, rs2289046, and rs754204). When these SNPs were modeled as a haplotype, the haplotype that included the G allele of rs2289046 was the only common haplotype associated with breast cancer (*P *= 0.0018). *IRS2 *rs2289046 and rs754204 were also associated with adult weight gain. No associations with breast cancer or with adiposity were found in the other genes included in this study: *ADRB2*, *ADRB3*, *GHRL*, *IRS1*, and *SHC1*.

## Abbreviations

BMI = body mass index; IRS = insulin receptor substrate; kb = kilobases; LD = linkage disequilibrium; OR = odds ratio; PCR = polymerase chain reaction; PMH = postmenopausal hormone; SNP = single nucleotide polymorphism.

## Competing interests

The authors declare that they have no competing interests.

## Authors' contributions

HSF established the study concept and design, had oversight over all aspects of the study, and contributed to manuscript writing. LRT participated in the design of the study, gene and SNP selection, assay design, data interpretation, and helped to draft the manuscript. WRD participated in SNP selection, carried out the statistical analysis, and helped to draft the manuscript. WT participated in assay design, sample preparation, genotyping, and helped to draft the manuscript. AVP participated in data interpretation and helped to draft the manuscript. VLS participated in gene selection and helped to draft the manuscript. EEC, as Principal Investigator in the Cancer Prevention Study II, provided the study population and helped to draft the manuscript. MJT helped to draft the manuscript. MB helped with study design, SNP selection, assay design, supervised all laboratory activities, and helped to draft the manuscript. All authors read and approved the final manuscript.

## Supplementary Material

Additional file 1 *ADBR2*, *ADBR3*, *GHRL*, *IRS1*, and *SHC1 *SNP associations with breast cancer. Click here for file
