# Supplementary material for: Genetic variation in candidate obesity genes ADRB2, ADRB3, GHRL, HSD11B1, IRS1, IRS2, and SHC1 and risk for breast cancer in the Cancer Prevention Study II
Source: Breast Cancer Res. 2008 Jul 8;10(4):R57. doi: 10.1186/bcr2114 (PMC2575528; doi:10.1186/bcr2114)
Supplement: Additional file 1 — ADBR2, ADBR3, GHRL, IRS1, and SHC1 SNP associations with breast cancer. [file bcr2114-S1.pdf]

Table 4. ADBR2, ADBR3, GHRL, IRS1, and SHC1 SNP associations with breast cancer

				Matching Adjusted[1]			Multivariate Adjusted[2]		
SNP	Alleles	Cases	Controls	Odds Ratio	95% CI	p-value[3]	Odds Ratio	95% CI	p-value[3]
ADRB2	rs877741								
	T/T	425	423	1.00	(-)		1.00	(-)	
	T/C	199	208	0.94	(0.74-1.19)		0.91	(0.71-1.16)	
	C/C	20	20	1.00	(0.52-1.91)	0.69	1.00	(0.51-1.94)	0.56
	T/C or C/C	219	228	0.95	(0.75-1.19)	0.64	0.92	(0.73-1.16)	0.49
	rs2400707								
	G/G	178	197	1.00	(-)		1.00	(-)	
	G/A	322	293	1.21	(0.94-1.57)		1.20	(0.92-1.56)	
	A/A	122	146	0.92	(0.67-1.27)	0.80	0.93	(0.67-1.28)	0.83
	G/A or A/A	444	439	1.12	(0.88-1.42)	0.37	1.11	(0.87-1.42)	0.40
	rs1042718								
	C/C	454	459	1.00	(-)		1.00	(-)	
	C/A	173	175	1.00	(0.78-1.28)		0.98	(0.76-1.26)	
	A/A	13	17	0.80	(0.38-1.66)	0.75	0.89	(0.42-1.88)	0.76
	C/A or A/A	186	192	0.98	(0.77-1.25)	0.88	0.97	(0.76-1.24)	0.81
	rs17108817								
	C/C	164	163	1.00	(-)		1.00	(-)	
	C/T	309	331	0.93	(0.72-1.22)		0.92	(0.70-1.20)	
	T/T	160	154	1.03	(0.75-1.40)	0.88	1.03	(0.75-1.41)	0.88
	C/T or T/T	469	485	0.96	(0.75-1.24)	0.78	0.95	(0.73-1.23)	0.70
ADRB3	rs4994								
	T/T	534	553	1.00	(-)		1.00	(-)	
	T/C	103	86	1.24	(0.91-1.69)		1.25	(0.91-1.72)	
	C/C	5	6	0.89	(0.27-2.92)	0.26	0.97	(0.28-3.31)	0.23
	T/C or C/C	108	92	1.22	(0.90-1.65)	0.20	1.23	(0.91-1.68)	0.19
GHRL	rs2075355								
	G/G	301	288	1.00	(-)		1.00	(-)	
	G/A	283	273	1.00	(0.79-1.27)		1.04	(0.82-1.32)	
	A/A	52	82	0.60	(0.41-0.88)	0.06	0.63	(0.42-0.93)	0.12
	G/A or A/A	335	355	0.91	(0.73-1.13)	0.40	0.94	(0.75-1.18)	0.61
	rs171336								
	G/G	235	263	1.00	(-)		1.00	(-)	
	G/T	315	270	1.30	(1.02-1.66)		1.26	(0.98-1.61)	
	T/T	75	101	0.82	(0.58-1.17)	0.96	0.75	(0.53-1.07)	0.56
	G/T or T/T	390	371	1.17	(0.93-1.47)	0.17	1.12	(0.89-1.41)	0.35
	rs35683								
	C/C	146	188	1.00	(-)		1.00	(-)	
	C/A	342	296	1.51	(1.16-1.97)		1.44	(1.10-1.90)	
	A/A	135	152	1.15	(0.84-1.59)	0.29	1.05	(0.76-1.46)	0.64
	C/A or A/A	477	448	1.39	(1.08-1.79)	0.01	1.31	(1.01-1.70)	0.04

Table 4. (continued)

SNP		Alleles	Cases	Controls	Matching Adjusted[1] Odds Ratio	95% CI	p-value[3]	Multivariate Adjusted[2] Odds Ratio	95% CI	p-value[3]
GHRL	rs4684677									
(cont.)		T/T	568	576	1.00	(-)		1.00	(-)	
		T/A	62	71	0.88	(0.61-1.26)		0.89	(0.61-1.28)	
		A/A	4	0	-	(*-*)	0.95	-	(*-*)	0.96
		T/A or A/A	66	71	0.93	(0.65-1.33)	0.69	0.94	(0.66-1.36)	0.76
	rs2075356									
		T/T	531	532	1.00	(-)		1.00	(-)	
		T/C	104	113	0.93	(0.69-1.24)		0.97	(0.72-1.30)	
		C/C	5	7	0.72	(0.23-2.30)	0.49	0.77	(0.24-2.50)	0.70
		T/C or C/C	109	120	0.91	(0.69-1.22)	0.54	0.96	(0.71-1.28)	0.76
	rs42451									
		C/C	342	344	1.00	(-)		1.00	(-)	
		C/T	264	252	1.06	(0.84-1.33)		1.09	(0.86-1.38)	
		T/T	36	55	0.65	(0.41-1.02)	0.34	0.65	(0.41-1.02)	0.43
		C/T or T/T	300	307	0.99	(0.79-1.23)	0.90	1.01	(0.80-1.26)	0.95
	rs35680									
		A/A	152	183	1.00	(-)		1.00	(-)	
		A/G	343	300	1.40	(1.07-1.83)		1.35	(1.03-1.78)	
		G/G	137	159	1.05	(0.77-1.44)	0.66	0.97	(0.70-1.34)	0.96
		A/G or G/G	480	459	1.28	(1.00-1.65)	0.05	1.22	(0.94-1.58)	0.12
	rs35679									
		G/G	389	376	1.00	(-)		1.00	(-)	
		G/A	224	228	0.96	(0.76-1.22)		0.98	(0.77-1.25)	
		A/A	32	48	0.63	(0.40-1.02)	0.15	0.60	(0.37-0.97)	0.14
		G/A or A/A	256	276	0.91	(0.73-1.13)	0.39	0.91	(0.73-1.15)	0.43
	rs696217									
		G/G	547	547	1.00	(-)		1.00	(-)	
		G/T	89	97	0.92	(0.67-1.26)		0.97	(0.70-1.33)	
		T/T	3	5	0.63	(0.15-2.64)	0.47	0.61	(0.14-2.63)	0.66
		G/T or T/T	92	102	0.91	(0.67-1.23)	0.53	0.95	(0.69-1.29)	0.75
	rs26802									
		T/T	315	299	1.00	(-)		1.00	(-)	
		T/G	281	278	0.96	(0.76-1.21)		0.97	(0.77-1.23)	
		G/G	51	78	0.63	(0.43-0.93)	0.06	0.60	(0.40-0.89)	0.05
		T/G or G/G	332	356	0.89	(0.72-1.11)	0.29	0.89	(0.71-1.11)	0.30
	rs27647									
		T/T	216	237	1.00	(-)		1.00	(-)	
		T/C	328	290	1.24	(0.97-1.58)		1.24	(0.96-1.59)	
		C/C	99	121	0.90	(0.65-1.24)	0.94	0.85	(0.61-1.19)	0.74
		T/C or C/C	427	411	1.14	(0.90-1.43)	0.27	1.12	(0.89-1.42)	0.34

Table 4 (continued)

SNP	Alleles	Cases	Controls	Matching Adjusted[1] Odds Ratio	Matching Adjusted[1] 95% CI	Matching Adjusted[1] p-value[3]	Multivariate Adjusted[2] Odds Ratio	Multivariate Adjusted[2] 95% CI	Multivariate Adjusted[2] p-value[3]
GHRL	rs26311								
(cont.)	G/G	490	508	1.00	(-)		1.00	(-)	
	G/C	140	136	1.08	(0.82-1.41)		1.12	(0.85-1.47)	
	C/C	9	8	1.15	(0.44-3.02)	0.55	1.02	(0.38-2.73)	0.48
	G/C or C/C	149	144	1.08	(0.83-1.40)	0.56	1.11	(0.85-1.45)	0.44
	rs27498								
	G/G	234	268	1.00	(-)		1.00	(-)	
	G/A	313	291	1.24	(0.98-1.57)		1.32	(1.03-1.68)	
	A/A	93	87	1.22	(0.86-1.71)	0.12	1.21	(0.85-1.71)	0.10
	G/A or A/A	406	378	1.24	(0.99-1.55)	0.07	1.29	(1.03-1.63)	0.03
	rs12630739								
	A/A	358	384	1.00	(-)		1.00	(-)	
	A/G	246	220	1.21	(0.96-1.53)		1.22	(0.96-1.55)	
	G/G	36	45	0.84	(0.53-1.34)	0.58	0.81	(0.50-1.30)	0.63
	A/G or G/G	282	265	1.15	(0.92-1.43)	0.23	1.15	(0.92-1.44)	0.23
IRS1	rs1801276								
	G/G	619	624	1.00	(-)		1.00	(-)	
	G/C	19	27	0.70	(0.38-1.27)	0.24	0.74	(0.40-1.36)	0.33
	rs1896832								
	A/A	527	538	1.00	(-)		1.00	(-)	
	A/G	108	108	1.03	(0.77-1.38)		1.02	(0.76-1.38)	
	G/G	6	5	1.24	(0.37-4.09)	0.75	1.49	(0.44-4.99)	0.67
	A/G or G/G	114	113	1.04	(0.78-1.39)	0.79	1.04	(0.78-1.40)	0.76
SHC1	rs4845401								
	C/C	223	224	1.00	(-)		1.00	(-)	
	C/G	318	314	1.02	(0.80-1.30)		1.03	(0.81-1.32)	
	G/G	99	114	0.87	(0.62-1.20)	0.50	0.86	(0.62-1.20)	0.51
	C/G or G/G	417	428	0.98	(0.78-1.23)	0.86	0.99	(0.78-1.25)	0.91

[1]Adjusted for race, birth date, and blood draw date.

[2]Adjusted for race, birth date, blood draw date, adult weight change, breast cysts, family history of breast cancer, and postmenopausal hormone use.

[3]p-values are provided for the log-additive model, and the dominant model.
ADRB2: beta-2-adrenergic receptor gene, ADRB3: beta-3-adrenergic receptor gene, GHRL: ghrelin gene, IRS1: insulin receptor substrate 1 gene, SHC1: Src homology 2 domain-containing transforming protein 1 gene, CI: confidence interval
